# Mendelian randomization to evaluate the causal relationship between liver enzymes and the risk of six specific bone and joint-related diseases

**DOI:** 10.3389/fimmu.2023.1195553

**Published:** 2023-08-16

**Authors:** Guiwu Huang, Wenchang Li, Yonglie Zhong, Weiming Liao, Zhiqi Zhang

**Affiliations:** ^1^ Department of Joint Surgery, The First Affiliated Hospital of Sun Yat-sen University, Sun Yat-sen University, Guangzhou, China; ^2^ Guangdong Provincial Key Laboratory of Orthopedics and Traumatology, The First Affiliated Hospital of Sun Yat-sen University, Sun Yat-sen University, Guangzhou, China; ^3^ Zhongshan School of Medicine, Sun Yat-sen University, Guangzhou, China

**Keywords:** Mendelian randomization, liver enzyme, bone and joint-related diseases, causality, genome-wide association studies

## Abstract

**Background:**

Studies of liver dysfunction in relation to bone and joint-related diseases are scarce, and its causality remains unclear. Our objective was to investigate whether serum liver enzymes are causally associated with bone and joint-related diseases using Mendelian randomization (MR) designs.

**Methods:**

Genetic data on serum liver enzymes (alkaline phosphatase (ALP); alanine transaminase (ALT); gamma-glutamyl transferase (GGT)) and six common bone and joint-related diseases (rheumatoid arthritis (RA), osteoporosis, osteoarthritis (OA), ankylosing spondylitis, psoriatic arthritis, and gout) were derived from independent genome-wide association studies of European ancestry. The inverse variance-weighted (IVW) method was applied for the main causal estimate. Complementary sensitivity analyses and reverse causal analyses were utilized to confirm the robustness of the results.

**Results:**

Using the IVW method, the positive causality between ALP and the risk of osteoporosis diagnosed by bone mineral density (BMD) at different sites was indicated (femoral neck, lumbar spine, and total body BMD, odds ratio (OR) [95% CI], 0.40 [0.23–0.69], 0.35 [0.19–0.67], and 0.33 [0.22–0.51], respectively). ALP was also linked to a higher risk of RA (OR [95% CI], 6.26 [1.69–23.51]). Evidence of potential harmful effects of higher levels of ALT on the risk of hip and knee OA was acquired (OR [95% CI], 2.48 [1.39–4.41] and 3.07 [1.49–6.30], respectively). No causal relationship was observed between GGT and these bone and joint-related diseases. The study also found that BMD were all negatively linked to ALP levels (OR [95% CI] for TBMD, FN-BMD, and LS-BMD: 0.993 [0.991–0.995], 0.993 [0.988–0.998], and 0.993 [0.989, 0.998], respectively) in the reverse causal analysis. The results were replicated via sensitivity analysis in the validation process.

**Conclusions:**

Our study revealed a significant association between liver function and bone and joint-related diseases.

## Highlights

- The genetically predicted alkaline phosphatase (ALP) was positively related to the risk of osteoporosis diagnosed by bone mineral density (BMD) and rheumatoid arthritis (RA).
- Genetic evidence of the potential harmful effects of higher levels of alanine transaminase (ALT) on the risk of hip and knee osteoarthritis was indicated.
- There was little evidence supporting causality between gamma-glutamyl transferase (GGT) and diverse bone and joint-related diseases.

## Introduction

Bones and joints play a vital role in supporting weight and facilitating movement in the human body. However, there has been a notable surge in the global prevalence of bone and joint-related diseases, with joint disease ranking as the second most prevalent cause of disability worldwide (1, 2). Hence, it is crucial to delve into the unexplored mechanisms underlying bone and joint-related diseases. Recent research has highlighted the importance of a signaling axis between the liver and bone, which is critical in maintaining body homeostasis by interacting with other organs like the gut, brain, and endocrine organs (3–6). Among patients with chronic liver disease, 5% to 20% also suffer from bone and joint-related diseases such as osteoporosis and fractures (7). A large retrospective study including 931,193 fracture patients found that fracture patients with chronic liver disease had significantly higher mortality and hospitalization rates than those without chronic liver disease. Another cross-sectional study including 17,476 adults found that the risk of osteoarthritis (OA) was 1.479 times higher in the presence of metabolic-associated fatty liver disease (8). A meta-analysis of 30 studies (29 retrospective cohort studies and one cross-sectional study) demonstrated that liver transplant patients were at five times greater risk for fractures than patients who had not received liver transplants (9). Hepatic osteodystrophy is defined as poor bone metabolism and structure resulting from chronic liver disease (10), and its underlying mechanisms may be related to immunity (11), growth factor (12), hormones (13), and nutrients (14).

Our aim was to comprehensively address aspects of bone and joint-related diseases within the constraints of our study. However, it is important to acknowledge that it is unavailable to address all the genetic bone and joint-related diseases due to the absence of a comprehensive genome-wide association studies (GWAS) database. We were unable to incorporate them into our investigation. Therefore, we opted to focus on six bone and joint-related diseases (rheumatoid arthritis (RA), osteoporosis, OA, ankylosing spondylitis (AS), psoriatic arthritis (PsA), and gout) for which there is publicly available high-quality GWAS data. The selection of these six bone and joint-related diseases with a high incidence rate was primarily driven by the objective of investigating the potential causal relationship between bone and joint-related diseases and liver enzymes. Existing reports indicate that genetics might contribute to this association (15, 16).

Liver enzymes such as alanine aminotransferase (ALT), alkaline phosphatase (ALP), and gamma-glutamyl transferase (GGT) are major biomarkers that reflect liver function, and the levels of blood liver enzymes are usually significantly elevated in patients with hepatic impairment (17–19). Notably, liver enzyme levels have also been found to be elevated in patients with bone and joint-related diseases. For example, serum ALP is elevated in patients with AS and RA, and serum ALT is elevated in patients with PsA (20–22). Thus, elevated blood liver enzymes may be considered a potential risk factor for bone and joint-related disease. However, little is known about the causal relationships between liver enzymes and bone and joint-related diseases.

Well-designed randomized controlled trials (RCTs)—the gold standard to imply causality—can effectively deal with potential confounders. However, RCTs take considerable time and might be impractical due to ethical concerns and financial limitations. As an important complementary causal research approach, Mendelian randomization (MR) uses genetic variants associated with the exposure as instrumental variables to robustly assess the causality between exposure and outcome, given that certain assumptions, including the absence of pleiotropy are valid (23). Against this background, the purpose of this study was to investigate the causal relationships between serum liver enzymes and six common bone and joint-related diseases via MR.

## Methods

### Study overview

**Figure 1:**
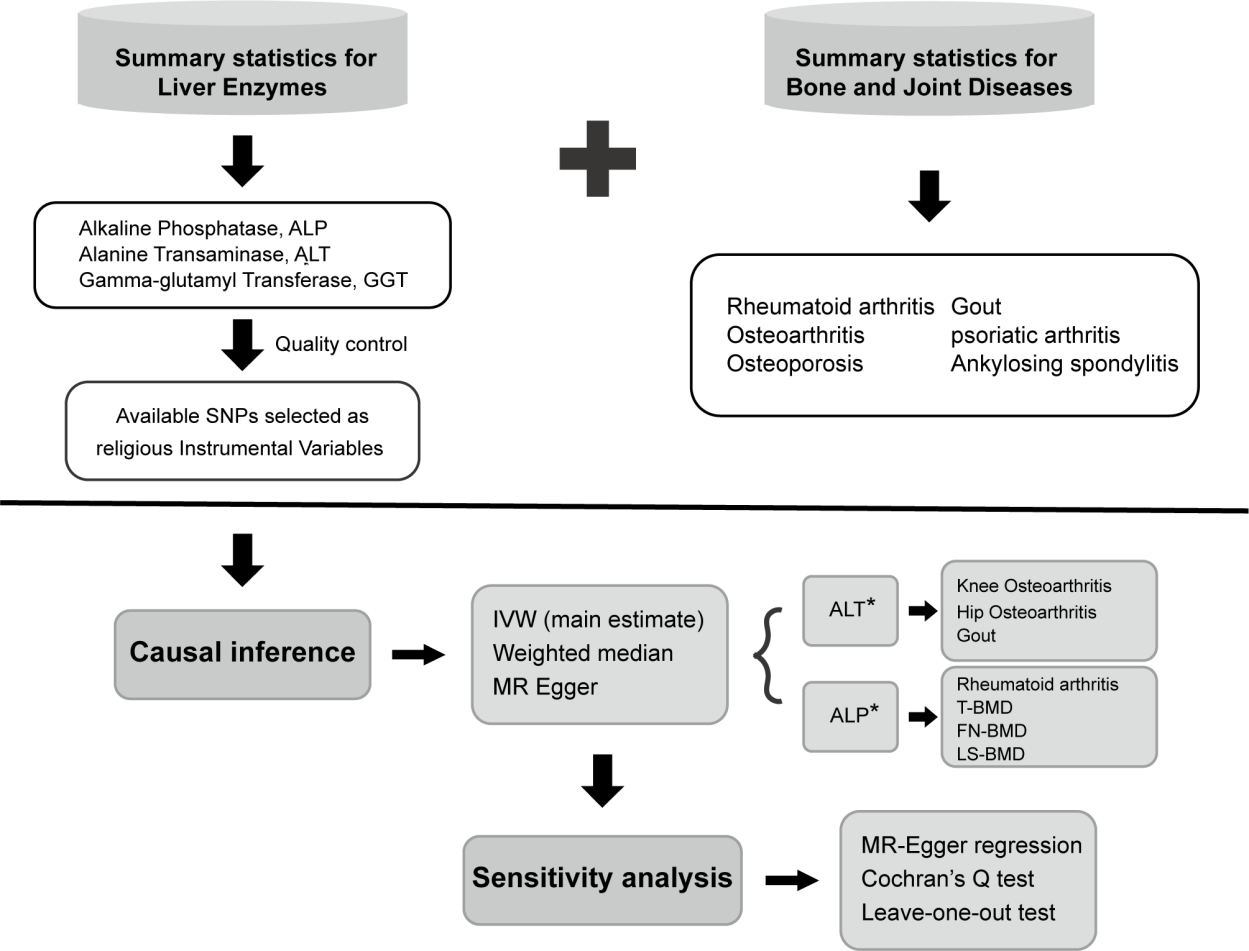
Overview and analysis process of our research. SNP, single-nucleotide polymorphism; IVW, inverse variance-weighted; MR, Mendelian randomization; ALT, alanine aminotransferase; ALP, alkaline phosphatase; OA, osteoarthritis; RA, rheumatoid arthritis; T-BMD, total body bone mineral density; FN-BMD, femoral neck bone mineral density; LS-BMD, lumbar spine bone mineral density.

An overview of the study design is shown in **Figure 1**. Three liver enzymes (ALT, ALP, and GGT) were regarded as exposure factors. Six bone and joint-related diseases, namely RA, osteoporosis, OA, AS, PsA, and gout, with 10 traits were considered the outcomes. The GWAS data of the exposure and outcome groups were collected. Using these data, MR analysis was performed to determine whether the liver enzymes have causal effects on the different bone and joint-related diseases. Three key assumptions must be met in a MR study: (1) the genetic variant is associated with the risk factor; (2) the genetic variant is not associated with confounders; and (3) the genetic variant influences the outcome only through the risk factor. All the data used in this study are published in GWAS databases; thus, additional ethical approval was not necessary. The study utilized various methods for statistical and sensitivity analysis, and the findings indicated that ALP was causally linked to decreased bone mineral density and a heightened risk of rheumatoid arthritis. Additionally, ALT was found to be causally associated with an increased risk of osteoarthritis and gout. However, GGT did not show any causal relation to the six bone and joint-related diseases studied.

### Data source

All the data utilized in this study was obtained from publicly available GWAS studies. The genetic analysis of liver enzymes was based on GWAS data for 753,010 European people from the UK Biobank and the Million Veteran Program (24). Osteoporosis was measured by bone mineral density (BMD). The data of total body BMD (T-BMD) were derived from a large GWAS meta-analysis involving about 66,628 European populations from the USA, Europe, and Australia (25). Femoral neck BMD (FN-BMD), lumbar spine BMD (LS-BMD), and forearm BMD (FA-BMD) data from a large whole genome sequencing, including 53,236 populations of European ancestry (26). BMD measurements were conducted using DXA according to standard manufacturer protocols. OA data were from the UK Biobank, including 77,052 cases and 378,169 controls (27). The diagnosis of OA was based on a radiological examination and clinical evaluation. The data for AS originates from the IEU OpenGWAS project database, with 1296 cases and 461,637 controls for the European population (28), and the data of RA comes from a large-scale GWAS meta-analysis involving 14,361 RA cases and 43,923 controls of European ancestry populations (29). The diagnosis of AS was defined by the code M16 in the International Classification of Diseases, Tenth Revision (ICD-10). All cases of RA met the 1987 criteria for RA diagnosis established by the American College of Rheumatology or were diagnosed by a professional rheumatologist. PsA data are from the FinnGen Biobank, involving 1,637 cases and 212,242 controls (30). The diagnosis of PsA also conformed to the ICD-10 code L40.5. Gout’s GWAS data come from a large GWAS meta-analysis involving 763,813 people (98.7% are of European ancestry) (31). Gout was identified based on serum urate levels, self-report, intake of urate-lowering medications, or the International Statistical Classification of Diseases and Related Health Problems (ICD) codes. The existing publications are listed in **Table 1**.

**Table 1:** GWAS information for bone and joint-related diseases was used in this study.

Trait	GWAS ID	Sample size/cases	PMID
Total body bone mineral density/TBMD	ebi-a-GCST005348	56,284	29304378
Femoral neck bone mineral density/FN-BMD	ie-a-980	32,735	26367794
Lumbar spine bone mineral density/LS-BMD	ie-a-982	28,498	26367794
Forearm bone mineral density/FA-BMD	ieu-a-977	8,143	26367794
Knee osteoarthritis	ebi-a-GCST007090	403,124/24,955	30664745
Hip osteoarthritis	ebi-a-GCST007091	393,873/15,704	30664745
Rheumatoid arthritis/RA	ieu-a-832	58,284/14,361	24390342
Ankylosing spondylitis/AS	ukb-b-18194	462,933/1,296	NA^a^
Psoriatic arthritis/PsA	finn-b-M13_PSORIARTH_ICD10	218,792/1,455	NA^a^
Gout	ebi-a-GCST008970	763,813/13,179	31578528

a GWAS data for ankylosing spondylitis were extracted from the UK Biobank, and GWAS data for psoriatic arthritis were extracted from the FinnGen research project.

### Selection of genetic instruments

According to previous research, for each liver enzyme trait, the single-nucleotide polymorphisms (SNPs) were then filtered using the following steps (32, 33): (1) A genome-wide significance threshold *p* < 5 × 10^−8^ was used. (2) Linkage disequilibrium (LD) test was performed using PLINK, and LD *r* ^2^ < 0.001 was adopted to ensure the independence of the selected genetic variants. For those variants missing from the outcome dataset, proxy SNPs with LD *r* ^2^ > 0.8 were used. (3) The F-statistic of each SNP was calculated, and SNPs with *F* < 10 were eliminated to avoid weak instrument bias. The proportion of variation explained (*R* ^2^) was also calculated to quantify the strength of genetic instruments with the following equation: *R* ^2^ = [2 × Beta^2^ × (1 − EAF) × EAF]/[2 × Beta^2^ × (1 − EAF) × EAF + 2 × SE^2^ × *N* × (1 − EAF) × EAF], where Beta indicates the genetic effect of each SNP, EAF is effect allele frequency, SE is the standard error, and *N* is the sample size. To assess the strength of the selected SNPs, the F-statistic was calculated using the following equation for each SNP: *F* = *R* ^2^ (*N* − *k* − 1)/*k*(1 − *R* ^2^), where *R* ^2^ represents the exposure variance explained by the selected SNPs, *N* is the sample size, and *k* represents the number of included instrumental variables. Weak instruments with F-statistics less than 10 were removed. The remaining independent instruments were used for subsequent MR analysis. (4) The SNPs that were incompatible or palindromic with intermediate allele frequencies were removed in the process of harmonizing. (5) Mendelian randomization pleiotropy residual sum and outlier test were performed to detect outliers and adjust for horizontal pleiotropy. If horizontal pleiotropy was detected among the instrumental variables, the outliers were removed.

### Statistical analyses

Each liver enzyme trait was matched with each bone and joint-related disease for the causal estimate. The inverse variance-weighted (IVW) method was first used to evaluate potential causal effects (34). The weighted median (WM) method (35) and the MR-Egger method (36) were then applied for sensitivity analyses. The causal effect estimates reflect the increase in outcome trait risk per 1-unit-higher standard deviation (SD) of each liver enzyme in the natural scale and presented as odds ratios (ORs) with their 95% confidence interval (CI). To correct for multiple comparisons for multiple hypotheses, the significance of causal inference (Bonferroni adjusted *p*-value) was set to less than 0.05/10 = 0.005 in the main IVW MR analyses (37).

Potential heterogeneity was quantified and tested by Cochran’s *Q* test (38), and directional pleiotropy was estimated by the MR-Egger regression test (36). Leave-one-out analysis was conducted to identify any potential outliers that independently influence the observed causal relationship. To eliminate the influence of confounding factors, we searched Phenoscanner (http://www.phenoscanner.medschl.cam.ac.uk/) to determine whether the selected SNPs are associated with other confounding risk factors. After excluding the confounder-related SNPs, we rechecked that the causal relationship remained significant. The statistical analyses above were mainly completed using the two-sample MR package (version 0.5.5) of R software (version 4.0.2). All reported *p*-values were two-tailed, and *p* < 0.05 was considered to indicate a significant difference.

### KEGG pathway enrichment analysis

To further explore the biological connection between liver enzymes and bone and joint-related diseases, we performed Kyoto Encyclopedia of Genes and Genomes (KEGG) enrichment analyses using the nearest genes for each lead SNP. Comprehensive gene list annotation and analysis were performed in Metascape (http://metascape.org/gp/index.html), a customer-friendly web-based portal. Enrichment dot bubbles were plotted using https://www.bioinformatics.com.cn, a free online platform for data analysis and visualization.

### Reverse causal relationship analysis

To investigate the potential reverse causal relationship between liver enzymes and specific bone and joint-related diseases, we conducted a reverse causal relationship analysis. Since there might be significant causality between liver enzymes and these diseases, we reversed the exposure and outcome. We followed the standard procedure to select appropriate genetic instruments and utilized IVW methods for the main analysis. Additionally, we performed sensitivity analyses to assess the robustness of our findings.

## Results

All the remaining SNPs and their related data, namely beta (β), standard error (SE), effect allele/other allele, and *p*-value, utilized to conduct the subsequent causal analysis are listed in **Supplementary File 1** (**Tables S1–S7**). Among them is effect size (β) for a SNP by modeling its association with the trait through a regression model, such as linear regression for a quantitative trait (each liver enzyme) or logistic regression for a qualitative outcome trait, assuming a linear trend per copy of an allele. The regression coefficients for quantitative traits (each liver enzyme) are presented with per 1-unit-higher SD, and for qualitative outcome traits, they are presented with per 1-unit-higher log odds, so that they are comparable across traits. The F-statistics of all the SNPs were greater than 10, indicating sufficient correlation strength between the instrumental variables and exposure traits.

**Figure 2:**
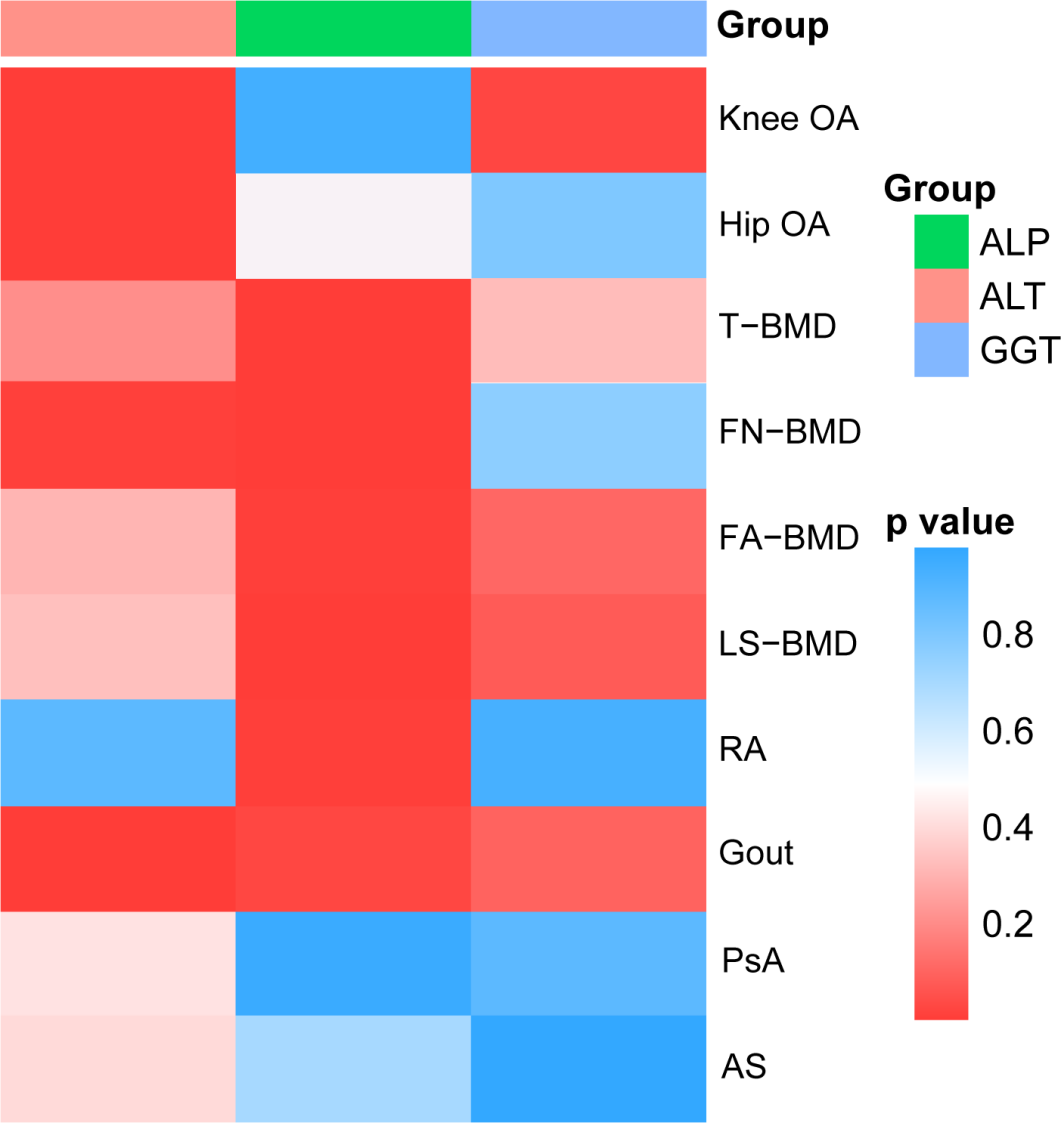
A panorama of all the liver enzyme features and their causal estimate on diverse phenotypes of bone and joint-related diseases identified at the nominal significance based on *p*-value of IVW methods. T-BMD, total body bone mineral density; FA-BMD, forearm bone mineral density; FN-BMD, femoral neck bone mineral density; LS-BMD, lumbar spine bone mineral density; OA, osteoarthritis; RA, rheumatoid arthritis; AS, ankylosing spondylitis; PsA, psoriatic arthritis; ALT, alanine aminotransferase; ALP, alkaline phosphatase; GGT, gamma-glutamyl transferase; IVW, inverse variance-weighted.

**Figure 3:**
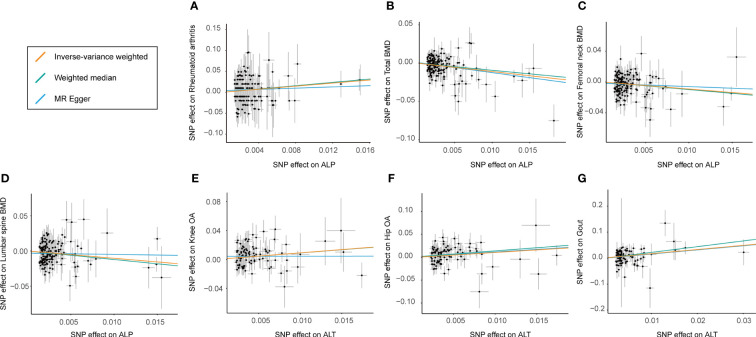
Scatter plot showing the effect sizes (beta) of the SNP effects on outcomes (*y*-axes) and the live enzymes (*x*-axes) with 95% confidence intervals. Each dot represents one of the SNPs used as the genetic instrument. The slopes indicate the estimate for each of the three different MR tests. SNP effects for each live enzyme are presented with per 1‐unit‐higher standard deviation (SD), and for outcome (diverse phenotypes of bone and joint-related diseases), they are presented with per 1‐unit‐higher log odds. **(A–D)** The estimated effect sizes of ALP on RA, total BMD, femoral neck BMD, and lumbar spine BMD. **(E–G)** SNP effects for estimated effect sizes of ALT on knee OA, hip OA, and gout. IVW, inverse variance-weighted; MR, Mendelian randomization; SNP, single-nucleotide polymorphism; RA, rheumatoid arthritis; BMD, body bone mineral density; ALP, alkaline phosphatase; ALT, alanine aminotransferase; OA, osteoarthritis.

The results of the preliminary analyses of the effects of genetically predicted liver enzymes on the risk of different bone and joint-related diseases are listed in **Supplementary File 2** (**Table S8**; **Figure 2**). Among them, a genetically predicted higher serum level of ALP was associated with a higher risk of RA (OR: 6.26, 95% CI: 1.69 to 23.15, *p* = 5.97 × 10^−3^). The positive causality between ALP and risk of osteoporosis diagnosed by bone mineral density (BMD) at different sites was indicated (TBMD, OR: 0.33, 95% CI: 0.22 to 0.51, *p* = 5.53 × 10^−7^; FN-BMD, OR: 0.40, 95% CI: 0.23 to 0.69, *p* = 8.44 × 10^−4^; LS-BMD, OR: 0.35, 95% CI: 0.19 to 0.67, *p* = 1.31 × 10^−3^). ALT showed positive causal relationships with the risk of OA and gout (knee OA, OR: 2.48, 95% CI: 1.39 to 4.41; *p* = 2.06 × 10^−3^; hip OA, OR: 3.07, 95% CI: 1.49–6.30; *p* = 2.27 × 10^−3^; gout, OR: 4.94, 95% CI: 1.98–12.37; *p* = 6.36 × 10^−4^). **Figure 3** shows a scatter plot of the causal estimates of the effects of each liver enzyme trait on the risk of different bone and joint-related diseases. No causal relationship could be supported based on our estimates between GGT and these bone and joint-related diseases.

To avoid excessive bias, a series of sensitivity analyses were conducted to test the reliability of the MR analysis and detect potential pleiotropy. As shown in **Table 2**, the intercept of the MR-Egger regression showed no sign of directional pleiotropy (*p* > 0.05) of all causality estimates. Cochran’s *Q* test indicated that considerable heterogeneity could not be excluded with confidence. The leave-one-out analysis conducted between ALP and gout detected three SNPs (rs174564, rs55714927, and rs6680628) that would drive the result, making the causal estimate unreliable. In the other leave-one-out analyses, no fundamental causal effects were changed, regardless of which SNP was removed, reflecting the robustness of our MR analysis (**Supplementary File 3**; **Supplementary Tables S9**–**S16**). As shown in **Table 3**, after removing the SNPs associated with confounding factors detected on Phenoscanner (listed in **Supplementary File 4**; **Supplementary Table S17**), the positive causal relationships found in primary analyses remained significant except the estimate between ALT and the risk of gout (OR (95% CI): 2.38 (0.77–7.34), *p* = 0.131, after adjustment), indicating that confounding factors are thought to be responsible for the finding between ALT and the risk of gout. MR power calculation showed strong power (100%) to detect a significant causal effect.

**Table 2:** Main causal relationships detected by MR analysis with different methods.

Liver enzymes	Traits	Nsnp	IVW		Weighted median		MR-Egger	
			OR (95% CI)	*p*-value	OR (95% CI)	*p*-value	OR (95% CI)	*p*-value
ALP	RA	154	6.26 (1.69, 23.15)	5.97 × 10^−3^	7.2 (1.02, 50.88)	0.048	2.09 (0.19, 23.47)	0.552
	TBMD	176	0.33 (0.22, 0.51)	5.53 × 10^−7^	0.39 (0.21, 0.72)	2.63 × 10^−3^	0.27 (0.12, 0.59)	1.24 × 10^−3^
	FN-BMD	166	0.40 (0.23, 0.69)	8.44 × 10^−4^	0.37 (0.16, 0.88)	0.024	0.66 (0.23, 1.84)	0.424
	LS-BMD	171	0.35 (0.19, 0.67)	1.31 × 10^−3^	0.3 (0.10, 0.85)	0.023	0.84 (0.26, 2.7)	0.765
ALT	Knee OA	99	2.48 (1.39, 4.41)	2.06 × 10^−3^	2.44 (1.13, 5.31)	0.024	1.01 (0.26, 4.03)	0.985
	Hip OA	99	3.07 (1.49, 6.30)	2.27 × 10^−3^	3.93 (1.38, 11.20)	0.010	3.09 (0.54, 17.5)	0.206
	Gout	96	4.94 (1.98, 12.37)	6.36 × 10^−4^	9.12 (2.75, 30.24)	2.98 × 10^−4^	4.89 (0.64, 37.34)	0.129

**Table 3:** Main causal relationships detected by MR analysis with compensated sensitivity methods.

Liver enzymes	Traits	Nsnp	Cochran’s *Q* test			MR-Egger regression		IVW*	
			*Q*	*p*-value	*I* ^2^	Egger intercept	*p*-value	OR (95% CI)	*p*-value
ALP	RA	154	184.77	0.041	16.1%	0.004	0.292	9.28 (2.43, 35.48)	1.13 × 10^−3^
	TBMD	176	276.30	1.60 × 10^−6^	35.9%	8.29 × 10^−4^	0.509	0.33 (0.21, 0.52)	1.70 × 10^−6^
	FN-BMD	166	222.56	1.88 × 10^−3^	25.0%	−1.74 × 10^−3^	0.275	0.48 (0.27, 0.86)	0.014
	LS-BMD	171	251.13	5.18 × 10^−5^	31.5%	−3.12 × 10^−3^	0.089	0.39 (0.20, 0.78)	7.36 × 10^−3^
ALT	Knee OA	99	128.95	0.020	22. 5%	4.21 × 10^−3^	0.166	2.64 (1.29, 5.39)	7.78 × 10^−3^
	Hip OA	99	125.14	0.033	20.1%	−3.05 × 10^−5^	0.994	2.83 (1.20, 6.66)	0.017
	Gout	96	155.01	9.97 × 10^−5^	37.4%	5.39 × 10^−5^	0.991	2.38 (0.77, 7.34)	0.131

KEGG pathway enrichment analysis indicated significant enrichment in the 20 most important regulation pathways (**Supplementary Figures S1**–**S6**). Several of these pathways may be involved in disease pathogenesis and are thus worthy of attention. Among them, RUNX2 transcriptional regulation and DNA methylation may be involved in the mechanism by which ALP reduces BMD and increases the risk of RA. Lipid homeostasis and circulatory system regulation involve multiple biological processes and molecular functions that may contribute to the essentiality of the association between ALT and the risk of OA.

In primary analysis, ALP was found to be causally associated with a higher risk of osteoporosis and RA, while ALT was associated with a higher risk of OA. In the reverse causal relationship analysis, we conducted IVW analyses to explore the potential causal effect of BMD on ALP and OA on ALT (**Supplementary File 5**; **Supplementary Table S18**). The results showed evidence for a causal effect of BMD on ALP levels. Specifically, BMD was inversely associated with ALP levels. The ORs and their corresponding 95% CIs were as follows: TBMD (OR: 0.993, 95% CI: 0.991 to 0.995, *p* = 1.06 × 10^−8^), FN-BMD (OR: 0.993, 95% CI: 0.988 to 0.998, *p* = 4.28 × 10^−3^), and LS-BMD (OR: 0.993, 95% CI: 0.989 to 0.998, *p* = 2.57 × 10^−3^) (**Table 4**). The estimates obtained using the weighted median method were consistent with these results. Furthermore, we conducted a MR-Egger regression analysis to assess directional pleiotropy. The intercepts of the MR-Egger regression indicated no evidence of directional pleiotropy for all causality estimates (*p* > 0.05) (**Table 5**). Although Cochran’s *Q* test suggested the presence of considerable heterogeneity, the leave-one-out analysis demonstrated that the fundamental causal effects remained unchanged regardless of the SNP removed, indicating the robustness of our MR analysis.

**Table 4:** Main causal relationships detected by MR analysis with different methods in revise causality analysis.

Exposure: traits	Outcome: liver enzymes	Nsnp	IVW		Weighted median		MR-Egger	
			OR (95% CI)	*p*-value	OR (95% CI)	*p*-value	OR (95%CI)	*p*-value
RA	ALP	28	1.001 (0.999, 1.002)	0.248	1.001 (0.999, 1.002)	0.350	1.000 (0.995, 1.006)	0.882
TBMD		57	0.993 (0.991, 0.995)	1.06 × 10^−8^	0.992 (0.989, 0.994)	6.09 × 10^−13^	0.991 (0.985, 0.997)	3.01 × 10^−3^
FN-BMD		13	0.993 (0.988, 0.998)	4.28 × 10^−3^	0.996 (0.992, 1.000)	0.050	0.998 (0.975, 1.022)	0.884
LS-BMD		12	0.993 (0.989, 0.998)	2.57 × 10^−3^	0.992 (0.989, 0.996)	1.04 × 10^−5^	0.989 (0.971, 1.007)	0.265
Knee OA	ALT	7	1.001 (0.988, 1.014)	0.862	1.003 (0.994, 1.012)	0.534	0.950 (0.894, 1.010)	0.159
Hip OA		19	1.000 (0.995, 1.004)	0.824	1.001 (0.998, 1.004)	0.510	0.998 (0.982, 1.013)	0.782

**Table 5:** Main causal relationships detected by MR analysis with compensated sensitivity methods in revise causality analysis.

Exposure: traits	Outcome: liver enzymes	Nsnp	Cochran’s *Q* test			MR-Egger regression	
			*Q*	*p*-value	*I* ^2^	Egger intercept	*p*-value
RA	ALP	28	70.187	1.05 × 10^−5^	61.5%	3.57 × 10^−5^	0.923
TBMD		57	187.830	3.84 × 10^−16^	70.2%	1.26 × 10^−4^	0.441
FN-BMD		13	51.485	7.65 × 10^−7^	76.7%	−3.52 × 10^−4^	0.638
LS-BMD		12	48.630	1.10 × 10^−6^	77.4%	2.99 × 10^−4^	0.660
Knee OA	ALT	7	49.048	7.29 × 10^−9^	87.8%	3.62 × 10^−3^	0.146
Hip OA		19	69.259	6.03 × 10^−8^	74.0%	1.72 × 10^−4^	0.822

## Discussion

The mechanisms underlying the elevated risk of bone and joint-related diseases in patients with liver disease are complex, and the precise mechanisms remain unknown. Our study provides strong genetic evidence in support of potential causal links between liver enzymes and an increased risk of specific bone and joint-related diseases based on a validated structured MR analysis. Specifically, we found that ALP was causally associated with a higher risk of osteoporosis and RA, while ALT was associated with a higher risk of OA. However, no causal relationships were observed between GGT and the diverse bone and joint-related diseases in our study. These results were significant in the main MR analyses and consistent across follow-up sensitivity analyses. These findings reveal causal relationships between liver enzymes and bone and joint-related diseases, suggesting that liver enzymes may serve as potential biomarkers for specific bone and joint-related diseases.

Serum ALP mainly comes from the liver and bones, with a minor amount coming from the intestine (39). Serum ALP is elevated when liver dysfunction develops (40). A study including 6,334 adults found that total serum ALP was inversely associated with BMD (41). Experimental high-ALP rat models established through bile-duct ligation exhibited skeletal fragility and impaired osteoblastogenesis, resulting in lower bone maximal force and stiffness (42). Naylor and Eastell found that increased ALP activity was associated with increased bone turnover (43). Interestingly, studies have shown that some liver cell membrane repair and protective agents can inhibit bone loss and promote bone formation (44, 45). For instance, ursodeoxycholic acid, another commonly used clinical drug for improving bile stasis, can promote mesenchymal stem cells to differentiate into osteoblasts, inhibit adipocyte differentiation, inhibit the production of inflammatory factors, and reduce inflammation-related side effects to promote bone formation (45, 46). These studies suggest that liver function improvement may regulate bone and joint-related metabolism. There are only a few clinical reports on the effect of ursodeoxycholic acid on bone mineral density. One of the studies pointed out that there was no significant effect on bone mineral density in patients with primary biliary cirrhosis (47). However, another study pointed out that the therapeutic effect of ursodeoxycholic acid on patients with primary biliary cirrhosis was related to the original vitamin D3 level of the patients, and the ALP level did not improve in patients with low vitamin D3 levels (48). Therefore, further comprehensive research is needed to understand the effect of ursodeoxycholic acid on the bone and joint systems of patients with hepatitis.

Studies of serum ALP concentration in relation to RA are scarce. In some observational studies, the ALP levels in the serum and synovia of RA patients are significantly elevated, and ALP is positively correlated with RA disease activity (49, 50). Meanwhile, patients with RA have high bone turnover and a high proportion of osteoporosis, which may explain the causal association between ALP and RA to some extent (49, 51).

The results of the KEGG analysis revealed other possible related mechanisms. RUNX2 is a classical regulatory transcription factor that promotes the differentiation of mesenchymal cells into osteoblasts (52–54). Interestingly, treatment with an ALP inhibitor reduced RUNX2 expression, a master transcriptional factor in osteoblasts, suggesting that the causal relationship between ALP and BMD may be driven through the RUNX2-associated signaling pathway (55). DNA methyltransferases can catalyze the methylation of DNACpG islands, thereby impairing genome stability and reducing the activity of gene transcription (56). DNA methylation has been shown to play an important role in the pathogenesis of osteoporosis (57–59). For example, the methylation of the osteoclast activation signal RANKL gene promoter region is significantly reduced in patients with osteoporosis, resulting in increased RANKL expression and increased osteoclast activity (60). In our study, ALP-related SNPs were enriched in DNA methylation pathways, suggesting that ALP may increase osteoclast activity by affecting DNA methylation levels. Further verified analyses should be applied in the future.

ALT is another important biomarker of liver function. Elevated serum ALT levels indicate impaired liver function (61). The relationship between ALT and OA has not been well investigated in epidemiology. However, our MR analysis suggested a definitive causal relationship between ALT and OA. We also obtained some hints from KEGG pathway analysis, suggesting that the mechanism may be related to circulatory system processes and lipid homeostasis. Circulation plays an important role in the pathogenesis of OA. Subchondral vascular hyperplasia, decreased arterial blood flow, and venous stasis have been reported in patients with knee osteoarthritis (62–64). Subchondral hyperemia in patients with OA leads to osteoblast hypoxia, alters the gene expression profile of cytokines, and ultimately leads to subchondral remodeling and chondrocyte degeneration, which are recognized as major components of the pathogenesis of OA (30, 65–68). Epidemiological studies suggest that ALT may significantly affect an individual’s circulatory system. For example, a retrospective study of 11,324 patients found that elevated plasma ALT levels were significantly associated with a higher risk of abnormal myocardial perfusion and myocardial infarction (69). The liver is the regulatory center of lipid metabolism in the body and maintains lipid homeostasis. In addition, serum desmosterol levels and the desmosterol/cholesterol ratio (a marker of cholesterol synthesis) were positively associated with ALT in a random population-based sample of 717 men (70). The concentration of cholesterol in synovial fluid is significantly elevated in individuals with OA (71). Choi et al. found that cholesterol and its metabolites directly activate retinoic acid-related orphan alpha receptors on chondrocytes, upregulate matrix-degrading enzymes, and increase the risk of OA (72). Notably, Maximos et al. demonstrated that the hepatic triglyceride content in patients with liver disease is positively correlated with the plasma ALT level (73). Based on a cohort study, Chen et al. found that the risk of new-onset metabolic fatty liver disease increased concurrently with increasing ALT levels (74). Hypertriglyceridemia is predominantly associated with worse pain in OA through central obesity (75). The relationship between ALT and the risk of OA could not be well established based on the present observational studies; thus, sufficient supporting evidence was needed from mechanical research.

The results of the reverse causal relationship suggested that BMD was inversely associated with ALP levels, but there was no reverse causal relationship between OA and ALT. These results were consistent across follow-up sensitivity analyses. Currently, no studies have clarified how BMD affects the liver enzyme. However, it is crucial to note that studies conducted in the last 10 years have revealed that the skeletal joint system is more than just a simple load-bearing structure. It is also a significant endocrine organ, and the cytokines it secretes govern numerous organs throughout the body, including the liver (76). For instance, a prospective analysis of 2,055 community populations discovered that women with lower levels of the typical bone-derived cytokine osteocalcin had a higher risk of nonalcoholic fatty liver disease, and further animal studies revealed that osteocalcin therapy for mice reduced hepatic steatosis (77). In addition, bone contains a lot of mesenchymal stem cells, and it has been discovered that these cells could regulate CD4 T-cell differentiation to lessen nonalcoholic liver steatosis in mouse models (78). Even though more direct studies are required, the aforementioned indirect findings may help partially explain the weak reverse causality relationship between BMD and ALP.

Our study has several limitations. First, there is an overlap between our disease GWAS data, like the presence of the UK Biobank in both the liver enzyme and OA databases. The challenge of resolving sample overlap across multiple GWAS databases remains unresolved due to the use of summary-level data that cannot be extracted individually. Certainly, it is important to exercise caution when drawing conclusions due to the potential inflation of causal associations resulting from sample overlap. Nevertheless, the strong instruments used in our study (i.e., F-statistic >> 10) could minimize potential bias due to the duplication of samples. Second, given that the summary-level statistics were obtained from the publicly available GWAS data, we could not perform other subgroup analyses to address associations with study-specific factors (e.g., age, gender, and other risk factors). The detailed characteristics like the effect allele frequency of genetic variants identified through GWAS study, can vary significantly across different ethnic backgrounds. These variations can influence the prevalence, genetic factors, and disease mechanisms of bone and joint-related diseases. Therefore, findings from a study conducted exclusively on European populations may not necessarily apply to other populations with distinct genetic backgrounds. Further research involving diverse populations is necessary to obtain a more comprehensive understanding of the genetic factors contributing to bone and joint-related diseases worldwide. Third, Cochran’s *Q* test indicated that considerable heterogeneity could not be excluded with confidence. However, based on the results of the IVW and weighted median methods, we believe that the robustness of our study was not biased by heterogeneity. Moreover, MR-Egger regression ruled out directional pleiotropy, indicating that heterogeneity did not cause pleiotropy or bias.

To our knowledge, this is the first study to comprehensively investigate the causal relationships between liver enzymes and bone and joint-related diseases based on publicly available genetic data via MR analysis. Although earlier studies have demonstrated that the risk of bone and joint-related illnesses will rise when liver function is poor for an extended period of time, no studies have yet suggested which specific liver function markers may raise the risk of certain bone and joint disorders. As a result, clinical recommendations are ambiguous, meaning that although medical professionals believe that patients with liver function impairment have an increased risk of developing bone and joint disorders, they are unsure of which specific bone and joint-related diseases the patient is more likely to develop. In this study, we discovered for patients with liver disease, blood ALP may be a potential biomarker of osteoporosis and RA, and blood ALT may be a potential biomarker of OA and gout. This finding strongly suggests the significant role of the liver in regulating bone and joint-related diseases. In patients with long-term liver disease, it is important to be mindful of screening for bone and joint-related diseases to facilitate early detection and treatment. The results may provide a new strategy to understand the relationships between liver and bone and guide clinical decisions related to disease management. Certainly, additional evidence is needed to further support this conclusion.

## Data availability statement

The original contributions presented in the study are included in the article/**Supplementary Material**. Further inquiries can be directed to the corresponding author.

## Author contributions

GH and WCL collected data, conducted the MR analysis, and wrote the manuscript. GH, WCL, and YZ contributed to the conceptualization, methodology, data acquisition and curation, formal analysis, visualization, writing, and editing. GH, WML, and ZZ contributed to the methodology, interpretation of data, writing, and editing. All authors reviewed the manuscript. ZZ takes full responsibility for the integrity of the study. All authors contributed to the article and approved the submitted version.

## Supplementary material

Supplementary Figure 1 KEGG pathway enrichment analysis of nearest genes for single-nucleotide polymorphisms used on the causality inference for ALP on the risk of rheumatoid arthritis. **(A)** Enriched ontology clusters. Each cluster is represented in a single color and shown as a circle. **(B)** Enrichment dot bubble where count means enriched number of genes.

Supplementary Figure 2 KEGG pathway enrichment analysis of nearest genes for single-nucleotide polymorphisms used on the causality inference for ALP on the risk of total body BMD. **(A)** Enriched ontology clusters. Each cluster is represented in a single color and shown as a circle. **(B)** Enrichment dot bubble where count means enriched number of genes.

Supplementary Figure 3 KEGG pathway enrichment analysis of nearest genes for single-nucleotide polymorphisms used on the causality inference for ALP on the risk of femoral neck BMD. **(A)** Enriched ontology clusters. Each cluster is represented in a single color and shown as a circle. **(B)** Enrichment dot bubble where count means enriched number of genes.

Supplementary Figure 4 KEGG pathway enrichment analysis of nearest genes for single-nucleotide polymorphisms used on the causality inference for ALP on the risk of Lumbar spine BMD. **(A)** Enriched ontology clusters. Each cluster is represented in a single color and shown as a circle. **(B)** Enrichment dot bubble where count means enriched number of genes.

Supplementary Figure 5 KEGG pathway enrichment analysis of nearest genes for single-nucleotide polymorphisms used on the causality inference for ALT on the risk of knee OA. **(A)** Enriched ontology clusters. Each cluster is represented in a single color and shown as a circle. (B) Enrichment dot bubble where count means enriched number of genes.

Supplementary Figure 6 KEGG pathway enrichment analysis of nearest genes for single-nucleotide polymorphisms used on the causality inference for ALT on the risk of hip OA. **(A)** Enriched ontology clusters. Each cluster is represented in a single color and shown as a circle. **(B)** Enrichment dot bubble where count means enriched number of genes.

Supplementary Figure 7 Plots of “leave-one-out” analyses **(A–C)** for MR analyses of the reverse causal relationship between BMD and ALP.

## References

[1] Sebbag E Felten R Sagez F Sibilia J Devilliers H Arnaud L. The world-wide burden of musculoskeletal diseases: a systematic analysis of the World Health Organization Burden of Diseases Database. Ann Rheumatic Dis (2019) 78(6):844–8. doi: 10.1136/annrheumdis-2019-215142 30987966

[2] Shen Y Huang X Wu J Lin X Zhou X Zhu Z. The Global Burden of Osteoporosis, Low Bone Mass, and Its Related Fracture in 204 Countries and Territories, 1990-2019. Front Endocrinol (2022) 13:882241. doi: 10.3389/fendo.2022.882241 PMC9165055 35669691

[3] Wang F So KF Xiao J Wang H. Organ-organ communication: The liver's perspective. Theranostics (2021) 11(7):3317–30. doi: 10.7150/thno.55795 PMC7847667 33537089

[4] Emdin CA Khera AV Kathiresan S. Mendelian Randomization. Jama (2017) 318(19):1925–6. doi: 10.1001/jama.2017.17219 29164242

[5] Yan L Wei JA Yang F Wang M Wang S Cheng T. Physical Exercise Prevented Stress-Induced Anxiety via Improving Brain RNA Methylation. Adv Sci (Weinheim Baden-Wurttemberg Germany) (2022) 9(24):e2105731. doi: 10.1002/advs.202105731 PMC9404392 35642952

[6] Lu K Shi TS Shen SY Shi Y Gao HL Wu J. Defects in a liver-bone axis contribute to hepatic osteodystrophy disease progression. Cell Metab (2022) 34(3):441–57.e7. doi: 10.1016/j.cmet.2022.02.006 35235775

[7] Jeong HM Kim DJ. Bone Diseases in Patients with Chronic Liver Disease. Int J Mol Sci (2019) 20(17). doi: 10.3390/ijms20174270 PMC6747370 31480433

[8] Han AL. Association between metabolic associated fatty liver disease and osteoarthritis using data from the Korean national health and nutrition examination survey (KNHANES). Inflammopharmacology (2021) 29(4):1111–8. doi: 10.1007/s10787-021-00842-7 34269951

[9] Lim WH Ng CH Ow ZGW Ho OTW Tay PWL Wong KL. A systematic review and meta-analysis on the incidence of osteoporosis and fractures after liver transplant. Transplant Int (2021) 34(6):1032–43. doi: 10.1111/tri.13863 33835638

[10] Ehnert S Aspera-Werz RH Ruoß M Dooley S Hengstler JG Nadalin S. Hepatic Osteodystrophy-Molecular Mechanisms Proposed to Favor Its Development. Int J Mol Sci (2019) 20(10). doi: 10.3390/ijms20102555 PMC6566554 31137669

[11] Choi M Park S Yi JK Kwon W Jang S Kim SY. Overexpression of hepatic serum amyloid A1 in mice increases IL-17-producing innate immune cells and decreases bone density. J Biol Chem (2021) 296:100595. doi: 10.1016/j.jbc.2021.100595 33781747 PMC8086136

[12] Liu Z Han T Werner H Rosen CJ Schaffler MB Yakar S. Reduced Serum IGF-1 Associated With Hepatic Osteodystrophy Is a Main Determinant of Low Cortical but Not Trabecular Bone Mass. J Bone mineral Res (2018) 33(1):123–36. doi: 10.1002/jbmr.3290 PMC5771972 28902430

[13] Deng N Mallepally N Peng FB Kanji A Marcelli M Hernaez R. Serum testosterone levels and testosterone supplementation in cirrhosis: A systematic review. Liver Int (2021) 41(10):2358–70. doi: 10.1111/liv.14938 33966337

[14] Roman-Garcia P Quiros-Gonzalez I Mottram L Lieben L Sharan K Wangwiwatsin A. Vitamin B_12_-dependent taurine synthesis regulates growth and bone mass. J Clin Invest (2014) 124(7):2988–3002. doi: 10.1172/JCI72606 24911144 PMC4071367

[15] Mosca A Fintini D Scorletti E Cappa M Paone L Zicari AM. Relationship between non-alcoholic steatohepatitis, PNPLA3 I148M genotype and bone mineral density in adolescents. Liver Int (2018) 38(12):2301–8. doi: 10.1111/liv.13955 30176114

[16] Huang X Lv Y He P Wang Z Xiong F He L. HDL impairs osteoclastogenesis and induces osteoclast apoptosis via upregulation of ABCG1 expression. Acta Biochim Biophys Sin (2018) 50(9):853–61. doi: 10.1093/abbs/gmy081 30060101

[17] Giannini EG Testa R Savarino V. Liver enzyme alteration: a guide for clinicians. CMAJ (2005) 172(3):367–79. doi: 10.1503/cmaj.1040752 PMC545762 15684121

[18] Abdulla S Hussain A Azim D Abduallah EH Elawamy H Nasim S. COVID-19-Induced Hepatic Injury: A Systematic Review and Meta-Analysis. Cureus (2020) 12(10):e10923. doi: 10.7759/cureus.10923 33194489 PMC7657443

[19] Mohamed MF Wadhavkar N Elfanagely Y Marino D Beran A Abdallah M. Etiologies and Outcomes of Transaminase Elevation > 1000 IU/L: A Systematic Review and Meta-Analysis. Dig Dis Sci (2023) 68(7):2843–52. doi: 10.1007/s10620-023-07962-w 37184617

[20] Kang KY Hong YS Park SH Ju JH. Increased serum alkaline phosphatase levels correlate with high disease activity and low bone mineral density in patients with axial spondyloarthritis. Semin Arthritis Rheumatism (2015) 45(2):202–7. doi: 10.1016/j.semarthrit.2015.03.002 25895696

[21] Curtis JR Beukelman T Onofrei A Cassell S Greenberg JD Kavanaugh A. Elevated liver enzyme tests among patients with rheumatoid arthritis or psoriatic arthritis treated with methotrexate and/or leflunomide. Ann Rheumatic Dis (2010) 69(1):43–7. doi: 10.1136/ard.2008.101378 PMC2794929 19147616

[22] Nanke Y Kotake S Akama H Kamatani N. Alkaline phosphatase in rheumatoid arthritis patients: possible contribution of bone-type ALP to the raised activities of ALP in rheumatoid arthritis patients. Clin Rheumatol (2002) 21(3):198–202. doi: 10.1007/s10067-002-8285-4 12111623

[23] Boehm FJ Zhou X. Statistical methods for Mendelian randomization in genome-wide association studies: A review. Comput Struct Biotechnol J (2022) 20:2338–51. doi: 10.1016/j.csbj.2022.05.015 PMC9123217 35615025

[24] Pazoki R Vujkovic M Elliott J Evangelou E Gill D Ghanbari M. Genetic analysis in European ancestry individuals identifies 517 loci associated with liver enzymes. Nat Commun (2021) 12(1):2579. doi: 10.1038/s41467-021-22338-2 33972514 PMC8110798

[25] Medina-Gomez C Kemp JP Trajanoska K Luan J Chesi A Ahluwalia TS. Life-Course Genome-wide Association Study Meta-analysis of Total Body BMD and Assessment of Age-Specific Effects. Am J Hum Genet (2018) 102(1):88–102. doi: 10.1016/j.ajhg.2017.12.005 29304378 PMC5777980

[26] Zheng HF Forgetta V Hsu YH Estrada K Rosello-Diez A Leo PJ. Whole-genome sequencing identifies EN1 as a determinant of bone density and fracture. Nature (2015) 526(7571):112–7. doi: 10.1038/nature14878 PMC4755714 26367794

[27] Tachmazidou I Hatzikotoulas K Southam L Esparza-Gordillo J Haberland V Zheng J. Identification of new therapeutic targets for osteoarthritis through genome-wide analyses of UK Biobank data. Nat Genet (2019) 51(2):230–6. doi: 10.1038/s41588-018-0327-1 PMC6400267 30664745

[28] IEU OpenGWAS project. Available at: https://gwas.mrcieu.ac.uk/.

[29] Okada Y Wu D Trynka G Raj T Terao C Ikari K. Genetics of rheumatoid arthritis contributes to biology and drug discovery. Nature (2014) 506(7488):376–81. doi: 10.1038/nature12873 PMC3944098 24390342

[30] Tsai PH Lee HS Siow TY Wang CY Chang YC Lin MH. Abnormal perfusion in patellofemoral subchondral bone marrow in the rat anterior cruciate ligament transection model of post-traumatic osteoarthritis: a dynamic contrast-enhanced magnetic resonance imaging study. Osteoarthritis Cartilage (2016) 24(1):129–33. doi: 10.1016/j.joca.2015.07.015 26241778

[31] Tin A Marten J Halperin Kuhns VL Li Y Wuttke M Kirsten H. Target genes, variants, tissues and transcriptional pathways influencing human serum urate levels. Nat Genet (2019) 51(10):1459–74. doi: 10.1038/s41588-019-0504-x PMC6858555 31578528

[32] Tan JS Liu NN Guo TT Hu S Hua L. Genetically predicted obesity and risk of deep vein thrombosis. Thromb Res (2021) 207:16–24. doi: 10.1016/j.thromres.2021.08.026 34507265

[33] Wang W Tan JS Hua L Zhu S Lin H Wu Y. Genetically Predicted Obesity Causally Increased the Risk of Hypertension Disorders in Pregnancy. Front Cardiovasc Med (2022) 9:888982. doi: 10.3389/fcvm.2022.888982 35694671 PMC9175023

[34] Burgess S Small DS Thompson SG. A review of instrumental variable estimators for Mendelian randomization. Stat Methods Med Res (2017) 26(5):2333–55. doi: 10.1177/0962280215597579 PMC5642006 26282889

[35] Bowden J Davey Smith G Haycock PC Burgess S. Consistent Estimation in Mendelian Randomization with Some Invalid Instruments Using a Weighted Median Estimator. Genet Epidemiol (2016) 40(4):304–14. doi: 10.1002/gepi.21965 PMC4849733 27061298

[36] Burgess S Thompson SG. Interpreting findings from Mendelian randomization using the MR-Egger method. Eur J Epidemiol (2017) 32(5):377–89. doi: 10.1007/s10654-017-0255-x PMC5506233 28527048

[37] Curtin F Schulz P. Multiple correlations and Bonferroni's correction. Biol Psychiatry (1998) 44(8):775–7. doi: 10.1016/S0006-3223(98)00043-2 9798082

[38] Cohen JF Chalumeau M Cohen R Korevaar DA Khoshnood B Bossuyt PM. Cochran's Q test was useful to assess heterogeneity in likelihood ratios in studies of diagnostic accuracy. J Clin Epidemiol (2015) 68(3):299–306. doi: 10.1016/j.jclinepi.2014.09.005 25441698

[39] Haarhaus M Cianciolo G Barbuto S La Manna G Gasperoni L Tripepi G. Alkaline Phosphatase: An Old Friend as Treatment Target for Cardiovascular and Mineral Bone Disorders in Chronic Kidney Disease. Nutrients (2022) 14(10). doi: 10.3390/nu14102124 PMC9144546 35631265

[40] Ponsioen CY Chapman RW Chazouillères O Hirschfield GM Karlsen TH Lohse AW. Surrogate endpoints for clinical trials in primary sclerosing cholangitis: Review and results from an International PSC Study Group consensus process. Hepatol (Baltimore Md) (2016) 63(4):1357–67. doi: 10.1002/hep.28256 26418478

[41] Shu J Tan A Li Y Huang H Yang J. The correlation between serum total alkaline phosphatase and bone mineral density in young adults. BMC Musculoskeletal Disord (2022) 23(1):467. doi: 10.1186/s12891-022-05438-y PMC9118774 35585578

[42] Weinreb M Pollak RD Ackerman Z. Experimental cholestatic liver disease through bile-duct ligation in rats results in skeletal fragility and impaired osteoblastogenesis. J Hepatol (2004) 40(3):385–90. doi: 10.1016/j.jhep.2003.11.032 15123350

[43] Naylor K Eastell R. Bone turnover markers: use in osteoporosis. Nat Rev Rheumatol (2012) 8(7):379–89. doi: 10.1038/nrrheum.2012.86 22664836

[44] Tao ZS Li TL Wei S. Silymarin prevents iron overload induced bone loss by inhibiting oxidative stress in an ovariectomized animal model. Chemico-biol Interact (2022) 366:110168. doi: 10.1016/j.cbi.2022.110168 36087815

[45] Arai Y Park H Park S Kim D Baek I Jeong L. Bile acid-based dual-functional prodrug nanoparticles for bone regeneration through hydrogen peroxide scavenging and osteogenic differentiation of mesenchymal stem cells. J Control Release (2020) 328:596–607. doi: 10.1016/j.jconrel.2020.09.023 32946872

[46] Cha BH Jung MJ Moon BK Kim JS Ma Y Arai Y. Administration of tauroursodeoxycholic acid enhances osteogenic differentiation of bone marrow-derived mesenchymal stem cells and bone regeneration. Bone (2016) 83:73–81. doi: 10.1016/j.bone.2015.10.011 26499839

[47] Angulo P Therneau TM Jorgensen A DeSotel CK Egan KS Dickson ER. Bone disease in patients with primary sclerosing cholangitis: prevalence, severity and prediction of progression. J Hepatol (1998) 29(5):729–35. doi: 10.1016/S0168-8278(98)80253-5 9833910

[48] Guo GY Shi YQ Wang L Ren X Han ZY Guo CC. Serum vitamin D level is associated with disease severity and response to ursodeoxycholic acid in primary biliary cirrhosis. Aliment Pharmacol Ther (2015) 42(2):221–30. doi: 10.1111/apt.13244 25982180

[49] Wheater G Elshahaly M Naraghi K Tuck SP Datta HK van Laar JM. Changes in bone density and bone turnover in patients with rheumatoid arthritis treated with rituximab, results from an exploratory, prospective study. PloS One (2018) 13(8):e0201527. doi: 10.1371/journal.pone.0201527 30080871 PMC6078302

[50] Aida S. Alkaline phosphatase isoenzyme activities in rheumatoid arthritis: hepatobiliary enzyme dissociation and relation to disease activity. Ann Rheumatic Dis (1993) 52(7):511–6. doi: 10.1136/ard.52.7.511 PMC1005089 8102225

[51] Adami G Saag KG. Osteoporosis Pathophysiology, Epidemiology, and Screening in Rheumatoid Arthritis. Curr Rheumatol Rep (2019) 21(7):34. doi: 10.1007/s11926-019-0836-7 31123839

[52] Kim WJ Shin HL Kim BS Kim HJ Ryoo HM. RUNX2-modifying enzymes: therapeutic targets for bone diseases. Exp Mol Med (2020) 52(8):1178–84. doi: 10.1038/s12276-020-0471-4 PMC8080656 32788656

[53] Shu HS Liu YL Tang XT Zhang XS Zhou B Zou W. Tracing the skeletal progenitor transition during postnatal bone formation. Cell Stem Cell (2021) 28(12):2122–36.e3. doi: 10.1016/j.stem.2021.08.010 34499868

[54] Kim HJ Lee S Park JM Cho HB Park JI Park JS. Development of a three-layer consecutive gene delivery system for enhanced bone regeneration. Biomaterials (2021) 277:121104. doi: 10.1016/j.biomaterials.2021.121104 34478934

[55] Jo S Han J Lee YL Yoon S Lee J Wang SE. Regulation of osteoblasts by alkaline phosphatase in ankylosing spondylitis. Int J Rheumatic Dis (2019) 22(2):252–61. doi: 10.1111/1756-185X.13419 30415492

[56] Schübeler D. Function and information content of DNA methylation. Nature (2015) 517(7534):321–6. doi: 10.1038/nature14192 25592537

[57] Peng S Gao Y Shi S Zhao D Cao H Fu T. LncRNA-AK137033 inhibits the osteogenic potential of adipose-derived stem cells in diabetic osteoporosis by regulating Wnt signaling pathway via DNA methylation. Cell Prolif (2022) 55(1):e13174. doi: 10.1111/cpr.13174 34953002 PMC8780896

[58] Morris JA Tsai PC Joehanes R Zheng J Trajanoska K Soerensen M. Epigenome-wide Association of DNA Methylation in Whole Blood With Bone Mineral Density. J Bone Miner Res (2017) 32(8):1644–50. doi: 10.1002/jbmr.3148 PMC5615229 28394087

[59] Visconti VV Cariati I Fittipaldi S Iundusi R Gasbarra E Tarantino U. DNA Methylation Signatures of Bone Metabolism in Osteoporosis and Osteoarthritis Aging-Related Diseases: An Updated Review. Int J Mol Sci (2021) 22(8). doi: 10.3390/ijms22084244 PMC8072687 33921902

[60] Wang P Cao Y Zhan D Wang D Wang B Liu Y. Influence of DNA methylation on the expression of OPG/RANKL in primary osteoporosis. Int J Med Sci (2018) 15(13):1480–5. doi: 10.7150/ijms.27333 PMC6216050 30443169

[61] Montanari NR Ramírez R Aggarwal A van Buuren N Doukas M Moon C. Multi-parametric analysis of human livers reveals variation in intrahepatic inflammation across phases of chronic hepatitis B infection. J Hepatol (2022) 77(2):332–43. doi: 10.1016/S0168-8278(22)00762-0 35218813

[62] Aaron RK Racine JR Voisinet A Evangelista P Dyke JP. Subchondral bone circulation in osteoarthritis of the human knee. Osteoarthritis Cartilage (2018) 26(7):940–4. doi: 10.1016/j.joca.2018.04.003 29723635

[63] Su W Liu G Liu X Zhou Y Sun Q Zhen G. Angiogenesis stimulated by elevated PDGF-BB in subchondral bone contributes to osteoarthritis development. JCI Insight (2020) 5(8). doi: 10.1172/jci.insight.135446 PMC7205438 32208385

[64] Ching K Houard X Berenbaum F Wen C. Hypertension meets osteoarthritis - revisiting the vascular aetiology hypothesis. Nat Rev Rheumatol (2021) 17(9):533–49. doi: 10.1038/s41584-021-00650-x 34316066

[65] Arnoldi CC Linderholm H Müssbichler H. Venous engorgement and intraosseous hypertension in osteoarthritis of the hip. J Bone Joint Surg Br volume (1972) 54(3):409–21. doi: 10.1302/0301-620X.54B3.409 5053885

[66] Kiaer T Pedersen NW Kristensen KD Starklint H. Intra-osseous pressure and oxygen tension in avascular necrosis and osteoarthritis of the hip. J Bone Joint Surg Br volume (1990) 72(6):1023–30. doi: 10.1302/0301-620X.72B6.2246284 2246284

[67] Warren SM Steinbrech DS Mehrara BJ Saadeh PB Greenwald JA Spector JA. Hypoxia regulates osteoblast gene expression. J Surg Res (2001) 99(1):147–55. doi: 10.1006/jsre.2001.6128 11421617

[68] Aaron RK Racine J Dyke JP. Contribution of Circulatory Disturbances in Subchondral Bone to the Pathophysiology of Osteoarthritis. Curr Rheumatol Rep (2017) 19(8):49. doi: 10.1007/s11926-017-0660-x 28718064

[69] Yardeni D Toledano R Novack V Shalev A Wolak A Rotman Y. The Association of Alanine Aminotransferase Levels With Myocardial Perfusion Imaging and Cardiovascular Morbidity. J Cardiovasc Pharmacol Ther (2022) 27:10742484221074585. doi: 10.1177/10742484221074585 35077243 PMC8840806

[70] Simonen M Männistö V Leppänen J Kaminska D Kärjä V Venesmaa S. Desmosterol in human nonalcoholic steatohepatitis. Hepatology (2013) 58(3):976–82. doi: 10.1002/hep.26342 23447451

[71] Zhang K Ji Y Dai H Khan AA Zhou Y Chen R. High-Density Lipoprotein Cholesterol and Apolipoprotein A1 in Synovial Fluid: Potential Predictors of Disease Severity of Primary Knee Osteoarthritis. Cartilage (2021) 13(1_suppl):1465s–73s. doi: 10.1177/19476035211007919 PMC8808802 33870758

[72] Choi W-S Lee G Song W-H Koh J-T Yang J Kwak J-S. The CH25H–CYP7B1–RORα axis of cholesterol metabolism regulates osteoarthritis. Nature (2019) 566(7743):254–8. doi: 10.1038/s41586-019-0920-1 30728500

[73] Maximos M Bril F Portillo Sanchez P Lomonaco R Orsak B Biernacki D. The role of liver fat and insulin resistance as determinants of plasma aminotransferase elevation in nonalcoholic fatty liver disease. Hepatol (Baltimore Md) (2015) 61(1):153–60. doi: 10.1002/hep.27395 25145475

[74] Chen JF Qin Q Wu ZQ Yan S Song XQ Ding SY. [A cohort study on the correlation between alanine aminotransferase trajectories and new-onset metabolic fatty liver disease]. Zhonghua liu xing bing xue za zhi = Zhonghua liuxingbingxue zazhi (2022) 43(2):234–40. 10.3760/cma.j.cn112338-20210809-00621 35184490

[75] Pan F Tian J Cicuttini F Jones G. Metabolic syndrome and trajectory of knee pain in older adults. Osteoarthritis Cartilage (2020) 28(1):45–52. doi: 10.1016/j.joca.2019.05.030 31394191

[76] Zheng XQ Lin JL Huang J Wu T Song CL. Targeting aging with the healthy skeletal system: The endocrine role of bone. Rev Endocrine Metab Disord (2023) 24(4):695–711. doi: 10.1007/s11154-023-09812-6 37402956

[77] Xia M Rong S Zhu X Yan H Chang X Sun X. Osteocalcin and Non-Alcoholic Fatty Liver Disease: Lessons From Two Population-Based Cohorts and Animal Models. J Bone Miner Res (2021) 36(4):712–28. doi: 10.1002/jbmr.4227 33270924

[78] Wang H Wang D Yang L Wang Y Jia J Na D. Compact bone-derived mesenchymal stem cells attenuate nonalcoholic steatohepatitis in a mouse model by modulation of CD4 cells differentiation. Int Immunopharmacol (2017) 42:67–73. doi: 10.1016/j.intimp.2016.11.012 27889556

