# Supplementary figures and images for: Mendelian randomization to evaluate the causal relationship between liver enzymes and the risk of six specific bone and joint-related diseases
Source: Front Immunol. 2023 Aug 16;14:1195553. doi: 10.3389/fimmu.2023.1195553 (PMC10469508; doi:10.3389/fimmu.2023.1195553)

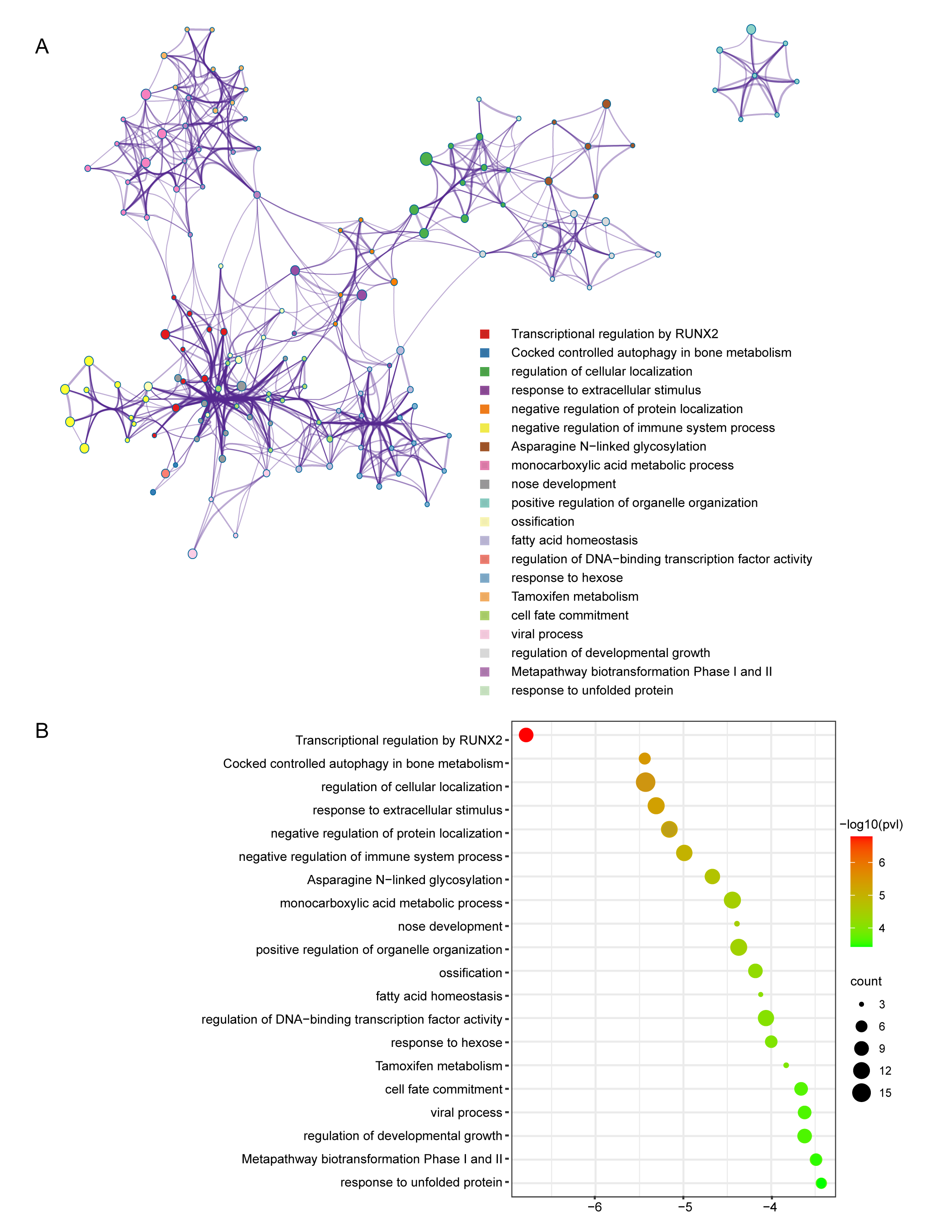

Supplement: Supplementary Figure 1 — KEGG pathway enrichment analysis of nearest genes for single-nucleotide polymorphisms used on the causality inference for ALP on the risk of rheumatoid arthritis. (A) Enriched ontology clusters. Each cluster is represented in a single color and shown as a circle. (B) Enrichment dot bubble where count means enriched number of genes. [file Image_1.tif]

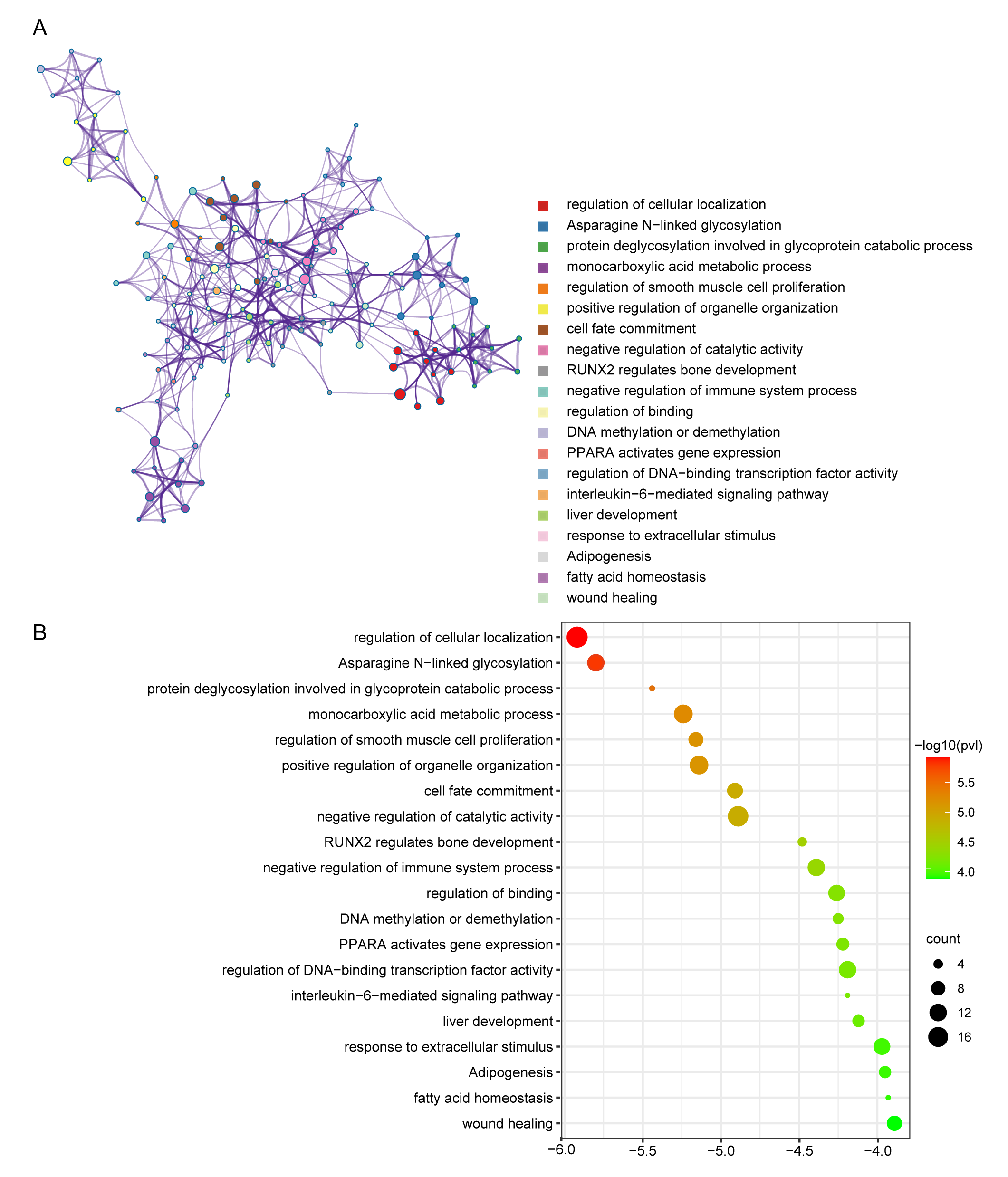

Supplement: Supplementary Figure 2 — KEGG pathway enrichment analysis of nearest genes for single-nucleotide polymorphisms used on the causality inference for ALP on the risk of total body BMD. (A) Enriched ontology clusters. Each cluster is represented in a single color and shown as a circle. (B) Enrichment dot bubble where count means enriched number of genes. [file Image_2.tif]

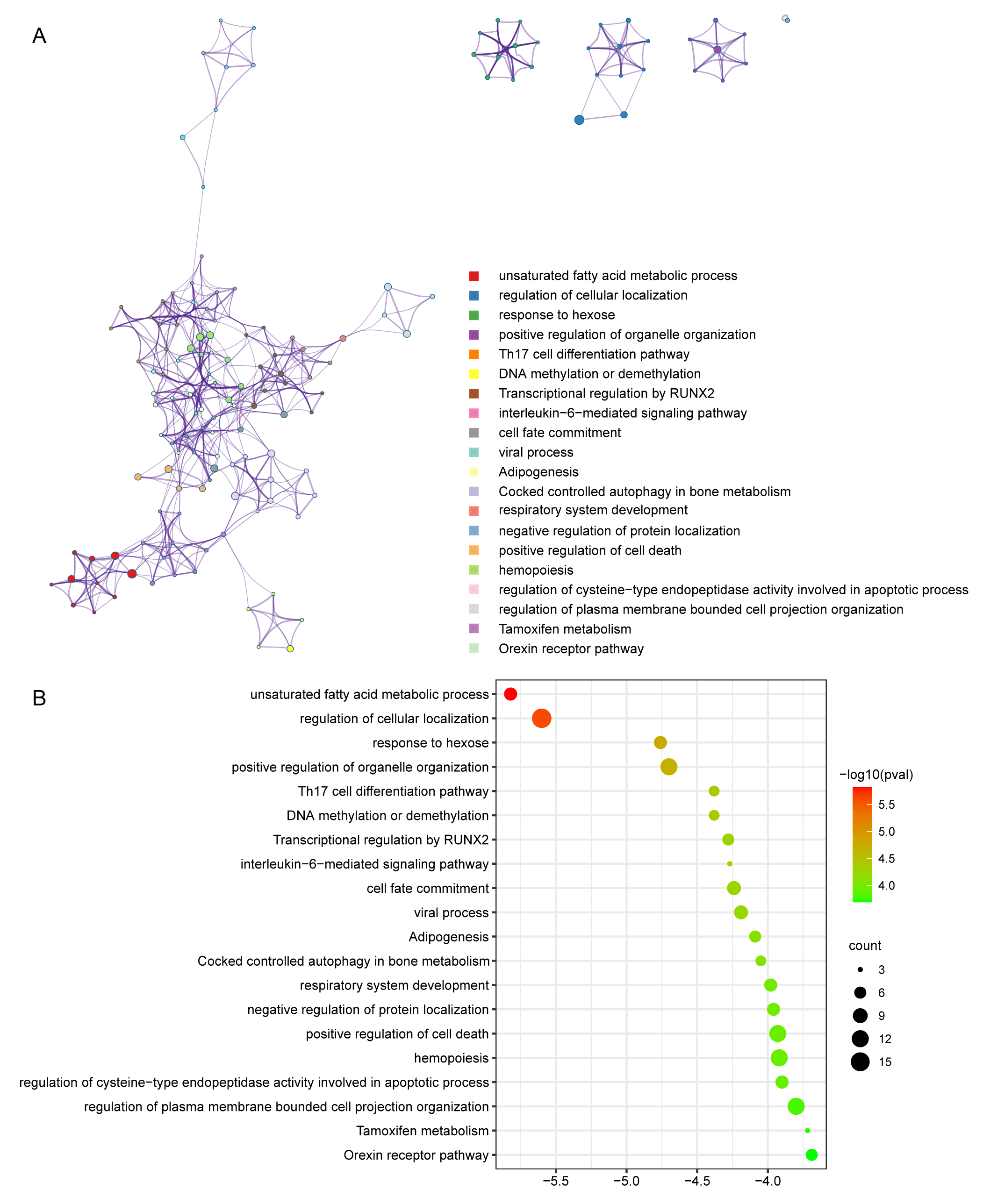

Supplement: Supplementary Figure 3 — KEGG pathway enrichment analysis of nearest genes for single-nucleotide polymorphisms used on the causality inference for ALP on the risk of femoral neck BMD. (A) Enriched ontology clusters. Each cluster is represented in a single color and shown as a circle. (B) Enrichment dot bubble where count means enriched number of genes. [file Image_3.tif]

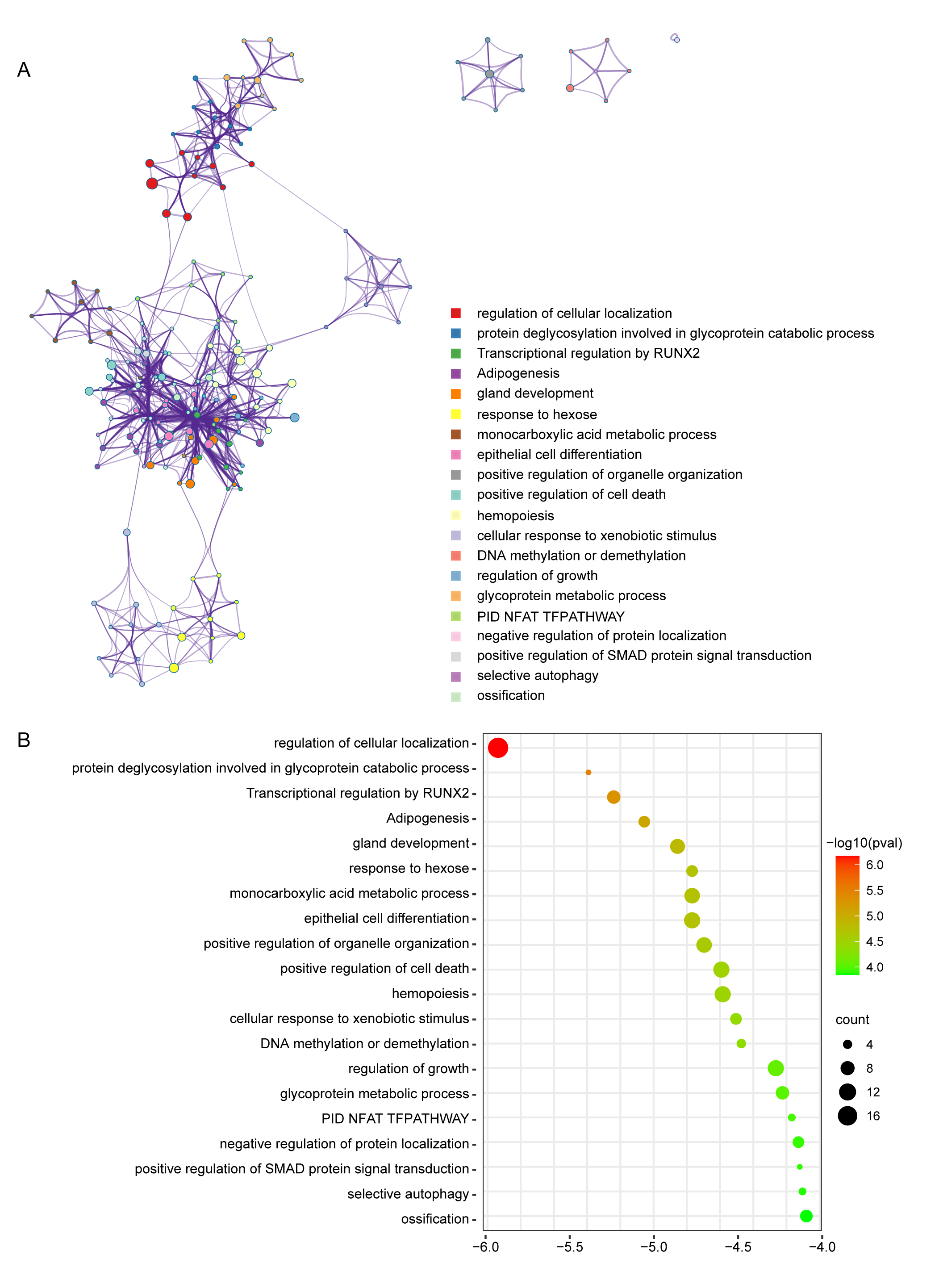

Supplement: Supplementary Figure 4 — KEGG pathway enrichment analysis of nearest genes for single-nucleotide polymorphisms used on the causality inference for ALP on the risk of Lumbar spine BMD. (A) Enriched ontology clusters. Each cluster is represented in a single color and shown as a circle. (B) Enrichment dot bubble where count means enriched number of genes. [file Image_4.tif]

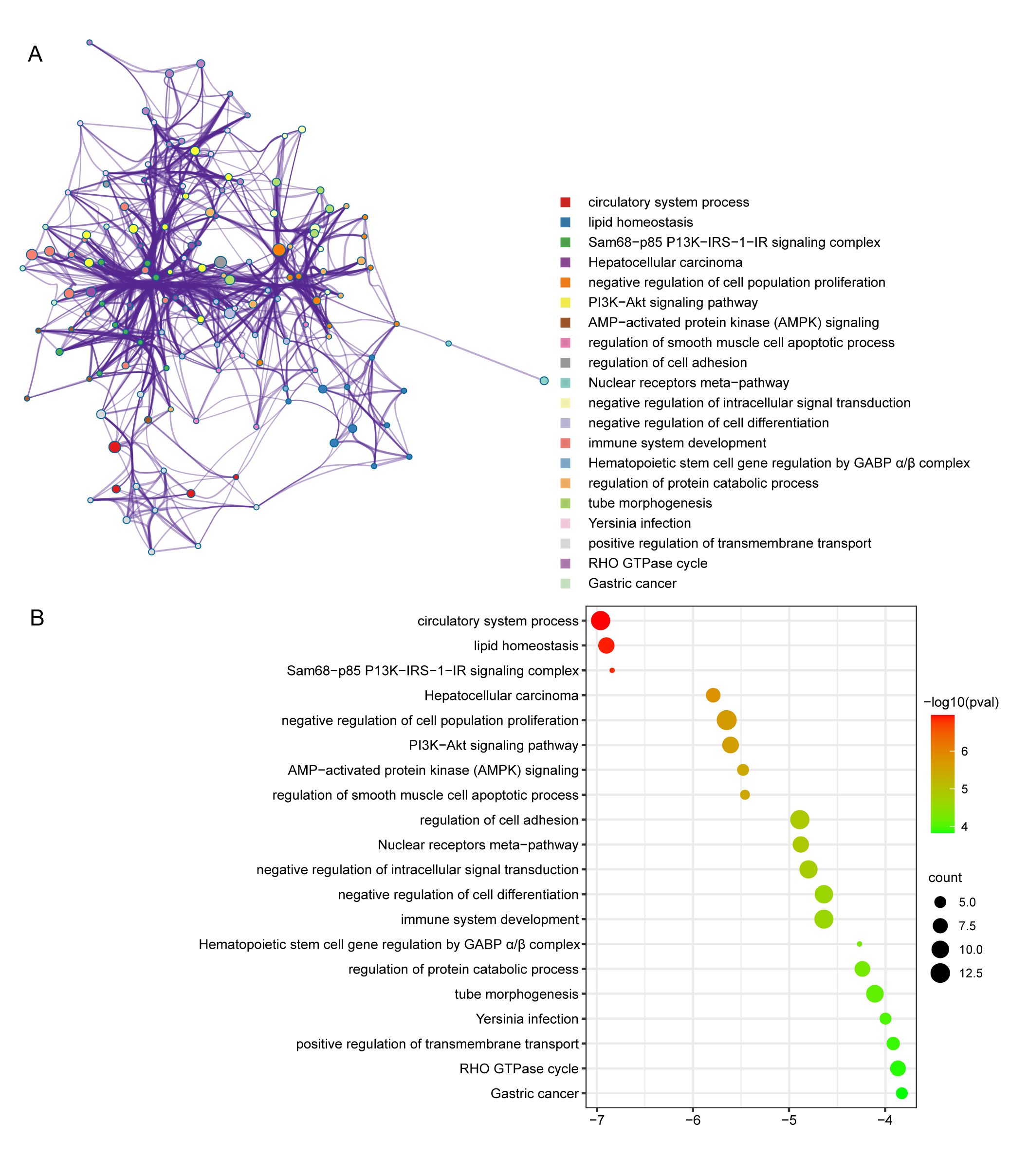

Supplement: Supplementary Figure 5 — KEGG pathway enrichment analysis of nearest genes for single-nucleotide polymorphisms used on the causality inference for ALT on the risk of knee OA. (A) Enriched ontology clusters. Each cluster is represented in a single color and shown as a circle. (B) Enrichment dot bubble where count means enriched number of genes. [file Image_5.tif]

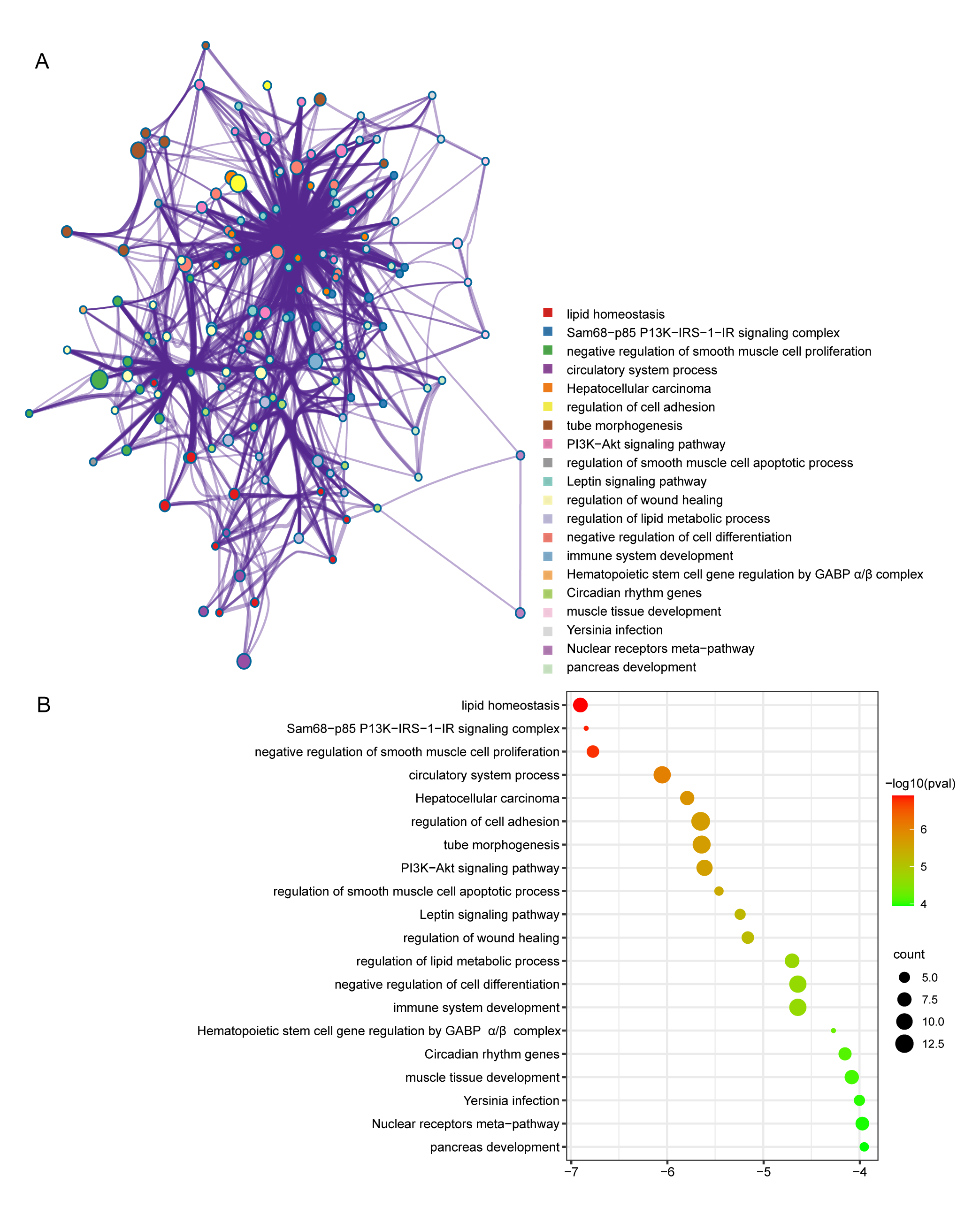

Supplement: Supplementary Figure 6 — KEGG pathway enrichment analysis of nearest genes for single-nucleotide polymorphisms used on the causality inference for ALT on the risk of hip OA. (A) Enriched ontology clusters. Each cluster is represented in a single color and shown as a circle. (B) Enrichment dot bubble where count means enriched number of genes. [file Image_6.tif]

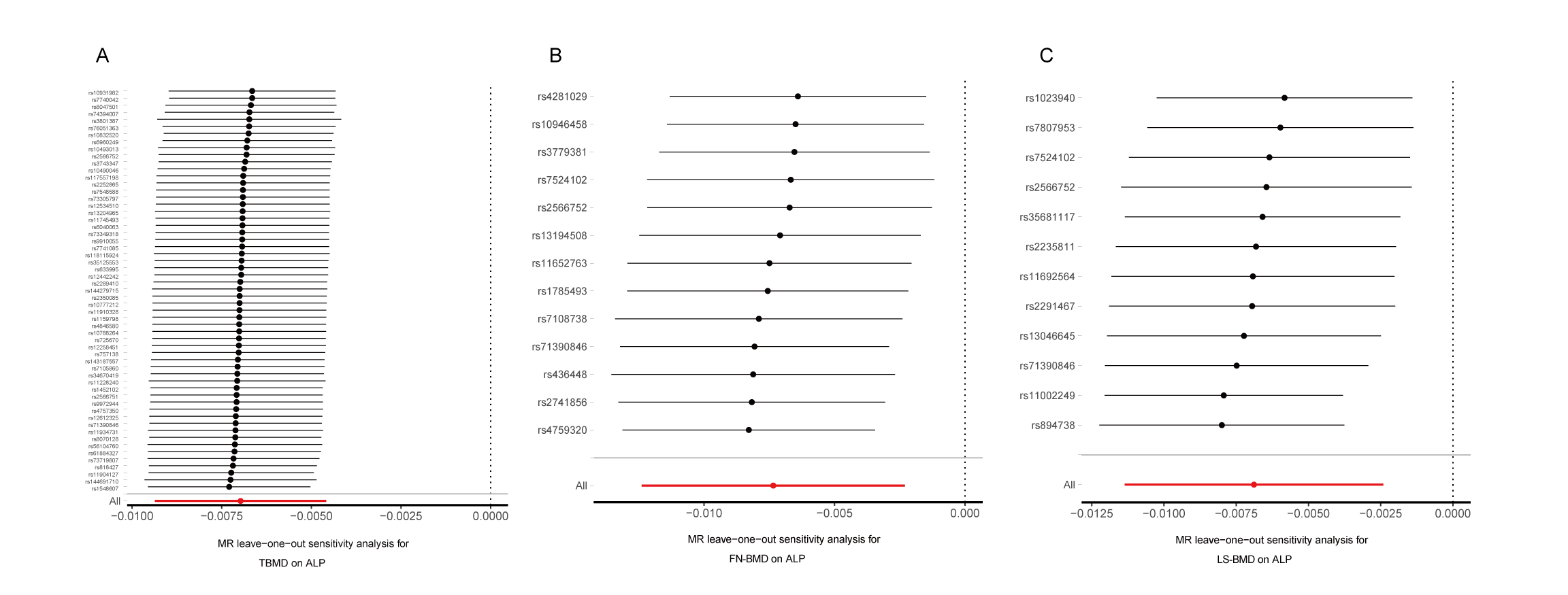

Supplement: Supplementary Figure 7 — Plots of “leave-one-out” analyses (A–C) for MR analyses of the reverse causal relationship between BMD and ALP. [file Image_7.tif]
